# Programmable RNA-Guided Large DNA Transgenesis by CRISPR/Cas9 and Site-Specific Integrase Bxb1

**DOI:** 10.3389/fbioe.2022.910151

**Published:** 2022-07-05

**Authors:** Vishnu Hosur, Benjamin E. Low, Michael V. Wiles

**Affiliations:** The Jackson Laboratory for Mammalian Genetics, Bar Harbor, ME, United States

**Keywords:** transgenesis, Cas9, Bxb1 integrase, recombinase, prime editing

## Abstract

The inability to insert large DNA constructs into the genome efficiently and precisely is a key challenge in genomic engineering. Random transgenesis, which is widely used, lacks precision, and comes with a slew of drawbacks. Lentiviral and adeno-associated viral methods are plagued by, respectively, DNA toxicity and a payload capacity of less than 5 kb. Homology-directed repair (HDR) techniques based on CRISPR-Cas9 can be effective, but only in the 1–5 kb range. In addition, long homology arms—DNA sequences that permit construct insertion—of lengths ranging from 0.5 to 5 kb are required by currently known HDR-based techniques. A potential new method that uses Cas9-guided transposases to insert DNA structures up to 10 kb in length works well in bacteria, but only in bacteria. Surmounting these roadblocks, a new toolkit has recently been developed that combines RNA-guided Cas9 and the site-specific integrase Bxb1 to integrate DNA constructs ranging in length from 5 to 43 kb into mouse zygotes with germline transmission and into human cells. This ground-breaking toolkit will give researchers a valuable resource for developing novel, urgently needed mouse and human induced pluripotent stem cell (hiPSC) models of cancer and other genetic diseases, as well as therapeutic gene integration and biopharmaceutical applications, such as the development of stable cell lines to produce therapeutic protein products.

## Significance of Large DNA Transgenesis

Researchers have discovered numerous links or correlations between putative structural variations (SVs) and gene expression and between SVs and phenotype, *via* long-read sequencing analysis of clinical samples and *via* large-scale collaboration programs such as ENCODE (The ENCODE Project Consortium, 2012). Some of the most common causes of cancer, including tumor suppressor genes (TSG) and oncogenic genes (ONC), originate at least in part from huge tandem duplications (TD) (Menghi et al., 2016; Willis et al., 2017; Menghi et al., 2018), as well as from kilobase-sized clusters of transcriptional enhancers, known as super-enhancers (SE) (Hnisz et al., 2013; Hnisz et al., 2015; Dave et al., 2017; Wang et al., 2019; Tang et al., 2020). However, researchers’ ability to study the role of large SVs in disease *via* model generation or synthetic biology is hindered by the inability to efficiently integrate large DNA constructions (greater than 10 kb) in mouse zygotes (Wang et al., 2013; Yang et al., 2013; Wang et al., 2015; Liu et al., 2019), mouse embryonic stem (mES) cells (Wang et al., 2015; Liu et al., 2019), or human cells (Chu et al., 2015; Jasin and Haber, 2016; Yang et al., 2020). Studies hindered by this barrier include: 1) generation of particular, potentially powerful mouse models [e.g., replacing a 100-kb region with its human equivalent requires at least three to 4 years (Wallace et al., 2007)], 2) identification of novel gene function when the gene is large, and 3) humanization of large regions of the mouse genome to study infectious diseases such as SARS-CoV-2, Ebola, and Hepatitis C virus. The need for an approach for insertion of large DNA constructs into the genome is highlighted by the current lack of an approach for effective modification of genome-wide DNA structures.

To meet the urgent requirement for approaches enabling large-DNA-construct integration, three distinct groups have collaborated to create a versatile and creative targeted transgenesis toolkit based on the Cas9-Bxb1 integrase system for accurate insertion of kb-sized DNA into the mammalian genome. Bxb1 integrase catalyzes effective and precise transgenesis by using DNA attachment sites (*attP* in the host genome and *attB* in the donor DNA, or *attB* in the host genome and *attP* in the donor DNA) as substrates (see Box 1). While alternative integrases such as phiC31, A118, SPBc, W, phiBT1, and phi370.1 are available, Bxb1 is the integrase of choice based on evidence that Bxb1, unlike these other integrases, has high efficiency and lacks pseudo sites in the mammalian genome. The new Cas9-Bxb1 toolkit will be useful for making important mouse models with large DNA inserts, such as potential new models for the Somatic Cell Genome Editing Consortium and humanized models of infectious illnesses, and will also be valuable for cell engineering and therapeutic gene therapy.

## Available Tools: Strengths and Weaknesses

Several approaches have been proposed to address some of the current genetic-engineering gaps, including random transgenesis, CRISPR/Cas9-mediated HDR, lentiviral-based and adeno-associated viruses (AAVs), and DNA transposases (e.g., Sleeping Beauty and piggyBac). However, none of these approaches address all of the gaps.

Random integration of transgenes is quick but ineffective (Haruyama et al., 2009; Laboulaye et al., 2018; Goodwin et al., 2019). It is also plagued by inherent variability as a result of complicated integrations at multiple locations, which can lead to segregation, position effects, and, in some cases, disruption of endogenous coding sequences, all of which complicate and hinder strain characterization. Furthermore, undocumented cassettes or even contaminated DNA pieces might be found in random insertion. Together, these haphazard insertions and modifications can result in phenotypic changes unrelated to function of the transgene.

While CRISPR/Cas9-mediated HDR of kb-sized DNA has been used to create transgenic mice and cell lines, this approach is unreliable. It has a variable and low success rate, especially when large (>10 kb) donor structures are required. Furthermore, while recent CRISPR/Cas9-based approaches, such as Easi (Efficient additions with ssDNA inserts)-CRISPR (using long single-stranded DNA donors) (Quadros et al., 2017), SPRINT (SHERLOCK-based profiling of *in vitro* transcription)-CRISPR (S-phase pronuclear injection of large DNA) (Abe et al., 2020), and 2C-HR (two-cell homologous recombination)-CRISPR (knock-in of large transgenes in two-cell stage embryos) (Gu et al., 2018), are highly efficient in the range of 1–6 kb, the efficiency of precise introduction of fragments greater than ∼7 kb in length is still not robust. The payload capacity of lentiviral-based techniques is superior (18 kb) (Chaudhari et al., 2020), but these techniques are linked to serious side effects such as genotoxicity (Montini et al., 2006) and immunogenicity (Nayak and Herzog, 2010). Although AAVs have few side effects, their payload capacity is less than 5 kb (Lai et al., 2010).

DNA transposases coupled with catalytically dead Cas9 for RNA-guided site-specificity have recently been designed to perform targeted transgenesis of up to 10 kb of DNA in bacteria (Munoz-Lopez and Garcia-Perez, 2010; Peters et al., 2017; Strecker et al., 2019; Vo et al., 2020). However, to date this system can be used only with prokaryotes. Furthermore, the significant off-target activity of transposases necessitates extensive engineering to accomplish efficient transgenesis. In addition, Sleeping Beauty and piggyBac, two of the most promising DNA transposons, are also linked to severe DNA toxicity (Wang et al., 2008; Tipanee et al., 2017).

In addition to the limitations discussed above, current approaches lack several other capabilities critical for efficient, versatile genetic engineering, including the ability to modify the genome sequentially to allow integration of additional kb-sized DNA constructs at a pre-existing locus (Brosh et al., 2021); control over copy numbers; control over integration orientation; DNA insertions without long kilobases of homology arms; and DNA insertions without traces of extraneous prokaryotic vector DNA. In sum, a key obstacle in genetic engineering remains the lack of sophisticated and economical techniques for precise integration of kb-sized DNA in human cells and animals. To overcome these limitations, a new toolkit—an RNA-guided Cas9-Bxb1 toolbox (Anzalone et al., 2021; Grandela et al., 2021; Ioannidi et al., 2021; Low et al., 2021)—has recently been developed that expands the breadth of precision transgenesis, genetic engineering, cell-based treatments, and synthetic biology.

## CRISPR/Cas9-Endonuclease With the Site-Specific Integrase Bxb1 in Mouse Zygotes

**FIGURE 1:**
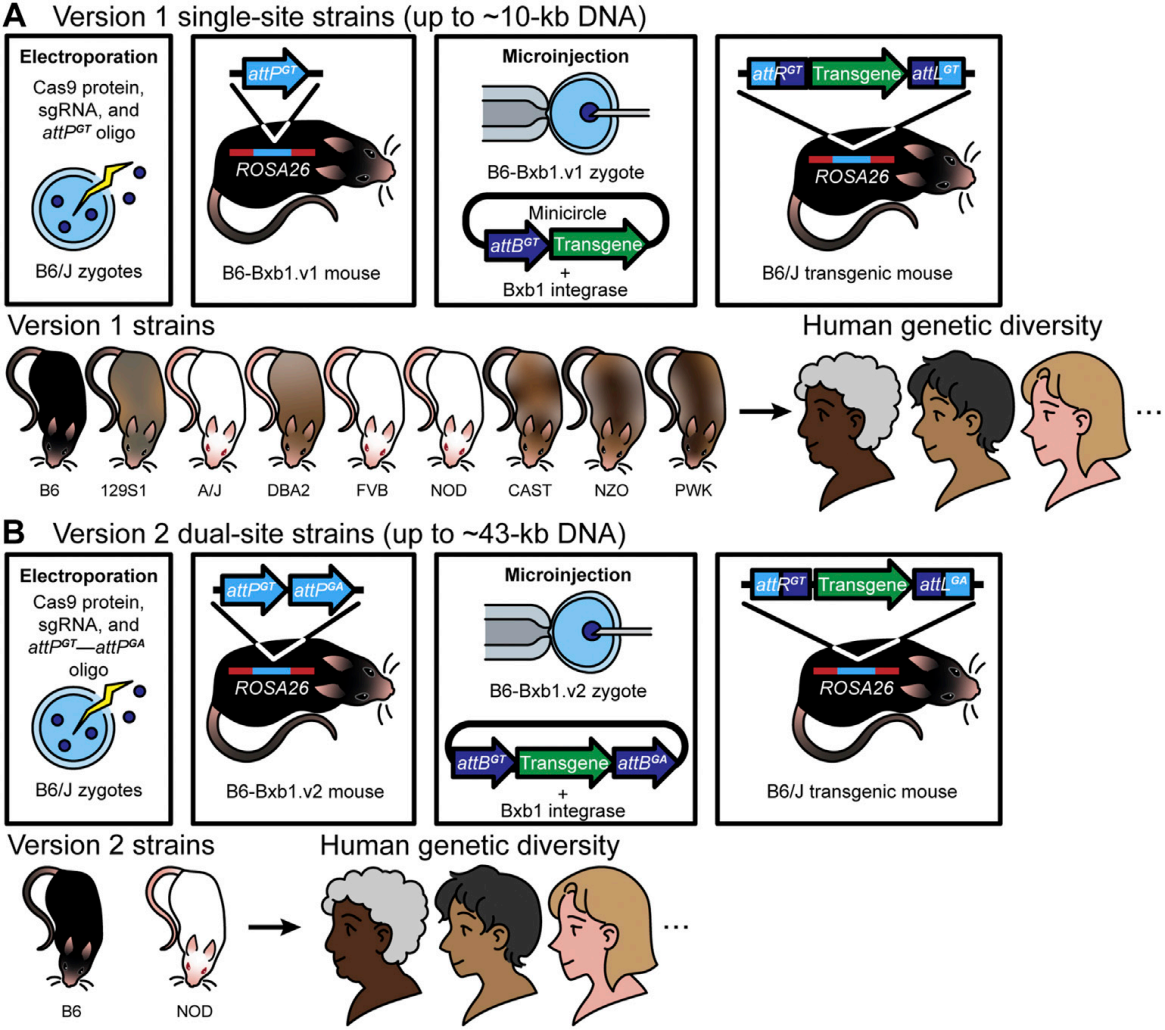
Version 1 (RMKI): **(A)** Over the past 5 years, we have generated mice using version 1 [the first-generation Cas9-Bxb1.v1 toolbox (a single *attP* ^*GT*^ site and a single *attB* ^*GT*^ site)] with successful targeted transgenesis of a series of DNA constructs up to 9.9 kb in length. The approach involves a) introduction of a single *attP* ^*GT*^ site into the safe harbor *ROSA*26 locus using electroporation in mouse zygotes by CRISPR/Cas9-mediated HDR **(A)**, and verification of insertion by PCR and Sanger sequencing, b) from those zygotes, establishment of a sequence-confirmed *attP* ^*GT*^ mouse strain—B6-Bxb1.v1 mice, c) pronuclear microinjection of *Bxb1* mRNA plus a large DNA construct (up to 9.9 kb) containing a single cognate *attB* ^*GT*^ site and the desired transgene into zygotes from matings of B6-Bxb1.v1 mice, for Bxb1 integrase-mediated insertion of DNA into the pre-placed *attP* ^*GT*^ site for creation of transgenic mice, and d) confirmation of the correct DNA insertion—recombinant attachment left (*attL*) and right (*attR*) sites—by PCR, Sanger sequencing, and Nanopore long-read sequencing, and subsequent appropriate expression of the transgene (top panel). Notably, because the genetic background of mice plays an important role in improving mouse-to-human translation (Rivera and Tessarollo, 2008; Reilly, 2016; Krupke et al., 2017), examining phenotypes in different genetic backgrounds can be a useful way to identify modifier genes and novel pathways. Towards this end, we have generated version 1 mice on different genetic backgrounds (bottom panel). Having the landing pad in more than one inbred mouse strain provides additional capabilities to study complex diseases, including cancer and neurodegenerative diseases. **(B)** Version 2 (RMCE): Building on the success of version 1 [the first-generation (Bxb1.v1) approach], we generated a Bxb1 version 2 (Cas9-Bxb1.v2) toolbox to further improve efficiency, and to enable insertion of even larger DNA constructs. The use of the dual heterologous attachment sites in the genome (*attP* ^*GA*^ and *attP* ^*GT*^), together with dual heterologous sites in the donor DNA (*attB* ^*GA*^ and *attB* ^*GT*^), enables exclusion of the vector backbone and allows insertion of longer tracts of DNA, as the *attB* ^*GA*^ and *attB* ^*GT*^ sites can be placed into any vector, including bacterial artificial chromosomes (BACs), simply by flanking the desired region to be integrated. This approach, commonly known as the recombinase-mediated cassette exchange (RMCE) approach, involves two crossover events between two heterologous or incompatible recombination sites flanking the donor DNA. This method can be used to exchange large transgene cassettes between the donor DNA and the genome. In Low et al., we provide evidence for insertion of a ∼43-kb construct (a threefold increase in size compared with Bxb1.v1) in B6-Bxb1.v2 mice (top panel). We have successfully generated version 2 mice on two different genetic backgrounds (bottom panel).

We recently developed an approach using the Bxb1 integrase to induce precise and efficient integration of large DNA constructs into the mouse genome (Low et al., 2021). Bxb1 uses *attP* and *attB* attachment sites to accomplish this DNA insertion. In a key innovative component of developing our approach, we pre-positioned an *attP* attachment site in the *ROSA26* safe harbor locus of several mouse strains, using CRISPR/Cas9-mediated HDR. We were then able to efficiently integrate large DNA constructs (∼5–∼43 kb) and generate single-copy transgenic mice. Below we describe two different versions of this recently developed approach for integrating large DNA constructs into a mouse safe harbor locus—version 1, or RMKI (recombinase mediated knock-in) (Figure 1A), for DNA constructs of up to 10 kb; and version 2, or recombinase-mediated cassette exchange (RMCE) (Figure 1B), for DNA constructs of up to ∼43 kb.

## One-Step Approach (One-Cell Stage, Electroporation and Two-Cell Stage, Microinjection) for rapid targeted transgenesis of large DNA constructs

Conventional transgenesis approaches require 18–24 months for generation of transgenic mice using the Bxb1 integrase; the first step involves insertion of an *attP* site in the mouse genome using CRISPR/Cas9-mediated HDR. After characterizing founder animals carrying that *attP* site, the mice must be backcrossed and then intercrossed to generate homozygous *attP* mice (9–12 months). Embryos from the homozygous *attP* mice are then microinjected with a large donor DNA construct containing the transgene and a cognate *attB* site, followed by backcrosses and intercrosses to generate homozygous transgenic mice (another 9–12 months). Our new approach, summarized below, eliminates these protracted steps by accomplishing both insertion of the *attP* site and microinjection of the DNA construct *in the same embryo*, thereby eliminating the generation of *attP* homozygous mice that is required in existing approaches and creating transgenic mice in one generation (9–12 months) instead of 18–24 months.

**FIGURE 2:**
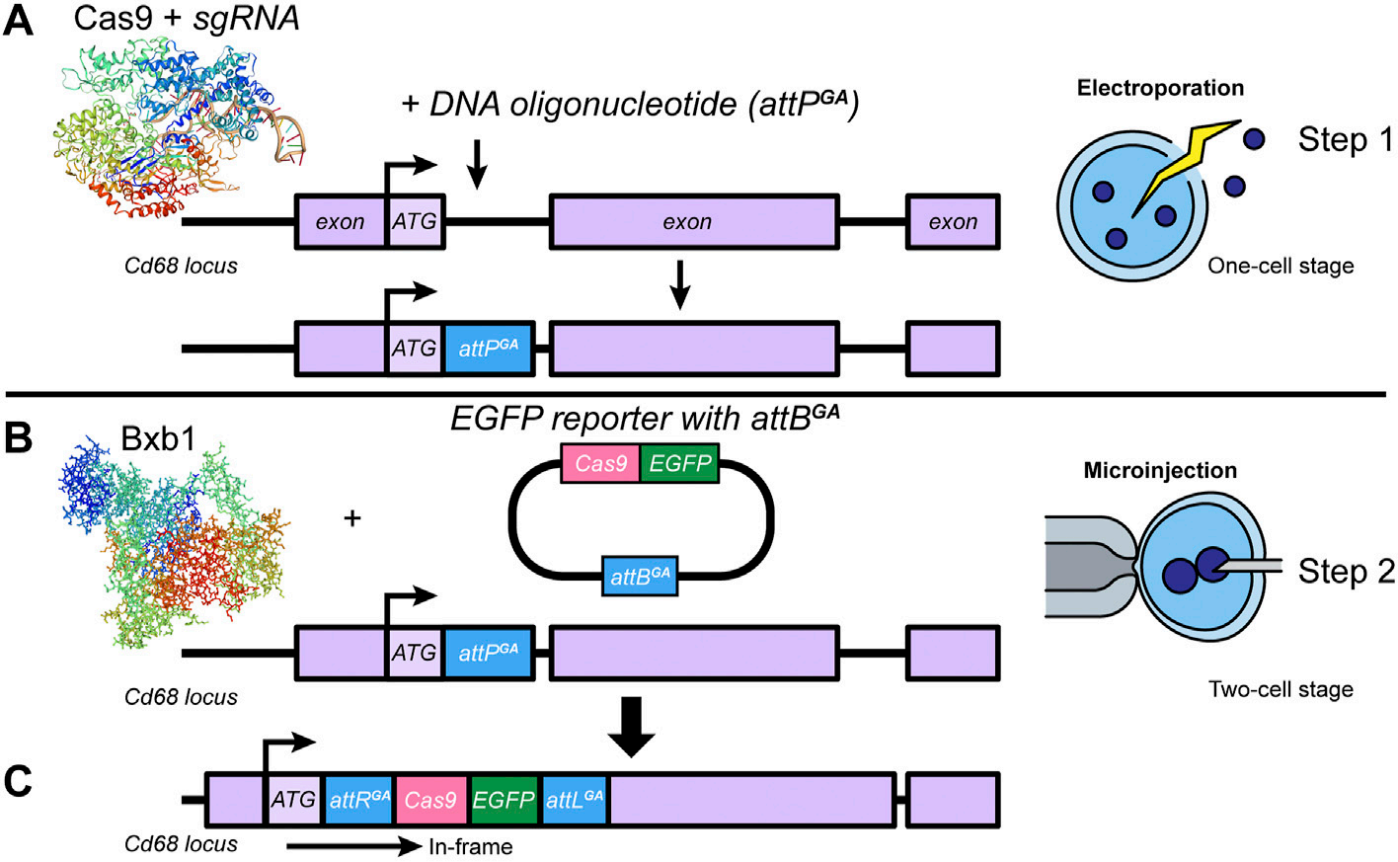
Bxb1-mediated targeted transgenesis of mouse zygotes in one-step. **(A)** At the zygote one-cell stage, using Cas9-mediated HDR, we inserted an *attP* ^*GA*^ site in the *Cd68* locus, adjacent to the start codon (ATG). **(B)** At the two-cell stage, in the same embryos, using Bxb1 we inserted a Cas9-EGFP reporter gene containing a cognate *attB* ^*GA*^ site. **(C)** The alleles with correct integration were used for germline transmission.

In the first step of our new approach, CRISPR/Cas9-meditated HDR in mouse zygotes *via* electroporation is used to insert, at the one-cell zygote stage, an *attP* site immediately downstream of the transcription initiation site ATG (Figure 2A). Then, in step 2, using the same embryos but at the two-cell stage, microinjection is used to introduce, in the presence of *Bxb1* mRNA, large donor DNA constructs carrying the transgene and the cognate *attB* site, allowing efficient, precise recombination of the donor DNA into the genomic *attP* site in a single generation (Figures 2B,C). Notably, electroporation is advantageous for both embryo survival and efficiency of HDR-mediated insertion of short oligos, whereas microinjection at the two-cell stage is essential for delivery of large DNA payloads. This novel one-step approach [one-cell stage, Electroporation (EP); and two-cell stage, Microinjection (MIJ)] not only significantly reduces the generation time, it, more importantly, enables a “plug-and-play” approach, i.e., one-step insertion of a large DNA construct in multiple mouse strains with no pre-placed attachment sites.

We used this innovative approach to successfully generate *Cd68-cas9* transgenic mice in one generation on the NSG background with 13% Bxb1-mediated integration efficiency. We sequence-confirmed that the *cas9* transgene was correctly integrated into the mouse *Cd68* endogenous locus. Homozygous mice are viable with no gross abnormalities.

## Targeted Nanopore Long-Read Sequencing for Efficient Validation of Correct Insertion of Transgenic Loci

**FIGURE 3:**
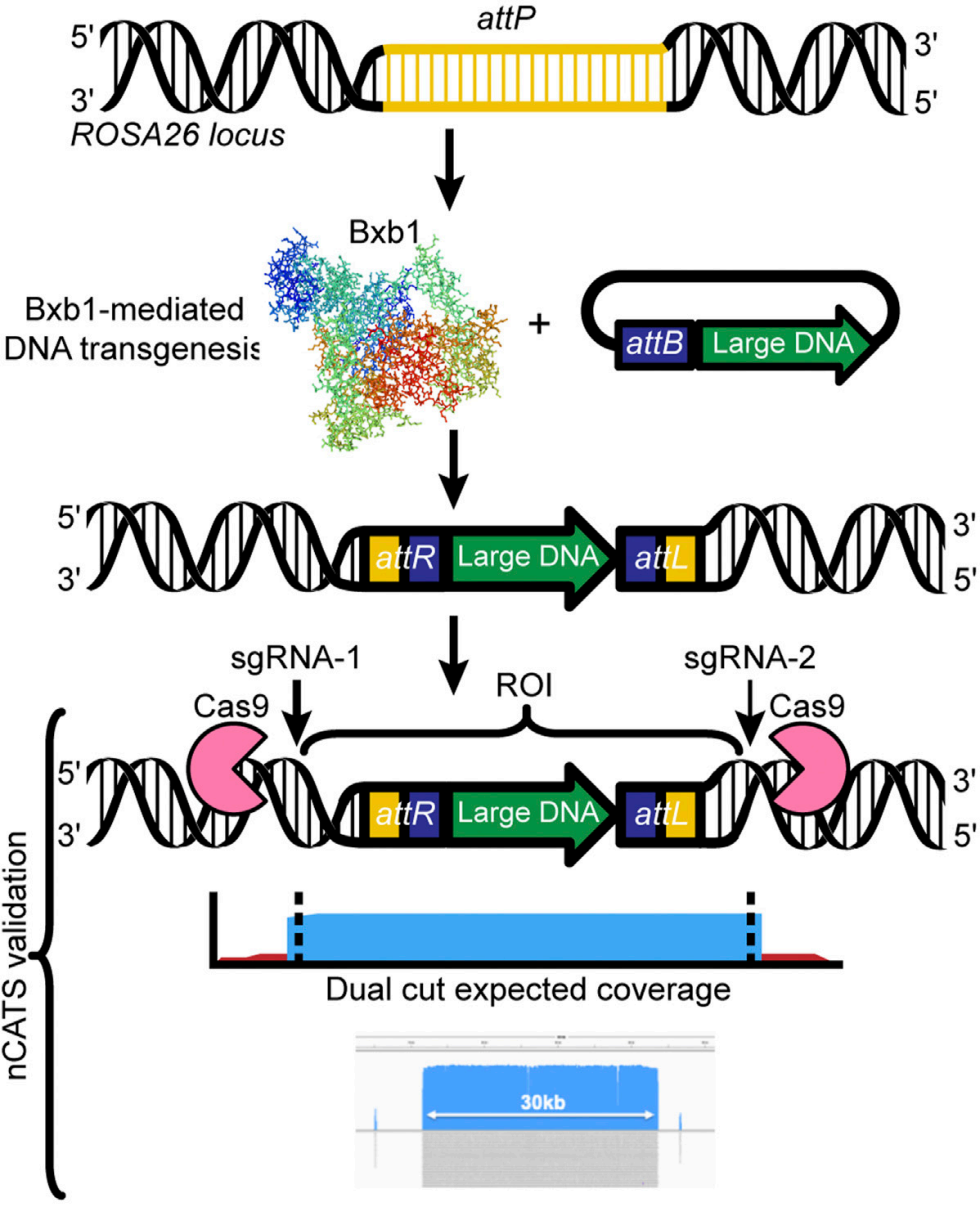
Designing a Cas9 targeted assay: Dual cut designs use a 5′ and 3′ sgRNA flanking the region of interest. The resulting library leads to generation of high coverage over the desired region (e.g., 5–30 kb), ideal for transgene validation. We have successfully used mouse ear notches to generate sufficient high molecular weight genomic DNA to sequence a 30-kb region up to 44x.

To ensure confirmation of correct insertion of transgenes, we have used two approaches: classical genotyping (PCR, Sanger sequencing) from DNA isolated from tail tissues, and Oxford Nanopore Technology’s Cas9-mediated amplification-free enrichment approach (Gilpatrick et al., 2020), a targeted sequencing approach. The Nanopore approach is relatively low-cost and can be applied to various starting materials, all while enriching regions of interest over native sequences. Importantly, neither of our two validation approaches requires sacrificing animals. Briefly, our workflow for the Nanopore targeted sequencing involves: 1) high molecular weight genomic DNA is extracted from mice carrying the transgene by using tissue from ear notches, 2) the region of interest is targeted by the Cas9-single guide RNA (sgRNA) complex and excised from the gDNA, 3) the resulting fragment is used to construct a Nanopore sequencing library without the need for amplification, and 4) upon sequencing, the region of interest is greatly enriched compared to the background gDNA (Figure 3). We have already validated the Nanopore targeted sequencing approach for transgenic inserts ranging from 5 to 43 kb in length (Low et al., 2021). Moreover, we enabled validation of not only the inserted transgene but also the regions bordering the two ends of the Bxb1 integration site (*attP* ^*GT*^ and *attP* ^*GA*^) in the *ROSA26* locus, by designing sgRNAs 2 kb upstream and downstream of the integration site.

## Twin Prime Editing + Bxb1

**FIGURE 4:**
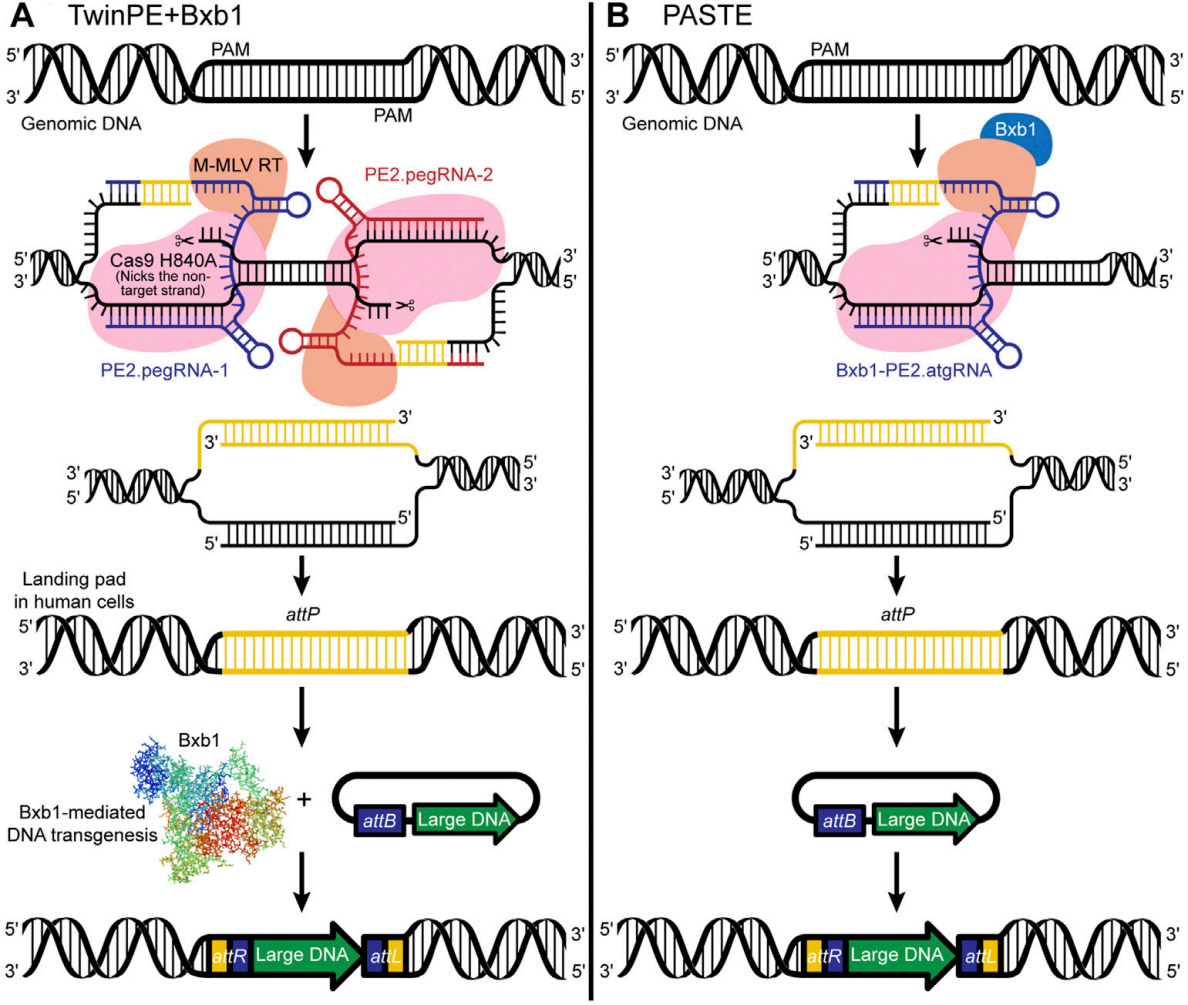
**(A)** TwinPE + Bxb1-mediated insertion of an *attP* sequence and creation of a landing pad in human cells for efficient insertion of large DNA sequences in the safe harbor locus. Whereas the previously described PE2 system uses a Cas9 nickase, an engineered MMLV-RT enzyme, and a pegRNA, the TwinPE uses two PE2 complexes that target two protospacer sequences on opposite strands. The TwinPE + Bxb1 system can make the pegRNA-encoded template with the desired insertion of short sequences (e.g., *attP* or *attB*) and replace the original DNA sequence. **(B)** PASTE is simply the PE2 system [a Cas9 nickase, an engineered MMLV-RT enzyme, and an attachment site-containing guide RNA (atgRNA) or pegRNA] fused with the Bxb1-integrase.

Twin Prime Editing (TwinPE) + Bxb1 enables targeted integration of large DNA plasmids (∼5.6 kb) at various safe-harbor loci in human cells (Anzalone et al., 2021) (see Box 2). Simultaneous delivery of a twinPE + Bxb1 complex with large donor plasmids enables integration at multiple genomic loci, permitting multiplexing capabilities. Notably, twinPE + Bxb1 mediates precise donor integration at the targeted site without causing inappropriate donor integration into the human genome (Figure 4A). Furthermore, because the modeling of large structural variants, including DNA inversions and rearrangements, involved in cancer and other diseases can be challenging, Anzalone et al. demonstrate that TwinPE + Bxb1 can facilitate inversions in human cells. The authors examined a ∼40-kb inversion between IDS and its pseudogene IDS2 to test the twinPE-Bxb1 inversion method on a therapeutically relevant locus. Inversions between these locations have been found in 13% of Hunter syndrome patients, and identification of the breakpoints in pathogenic alleles has showed that the inversion frequently occurs inside a recombination hotspot seen in both IDS and IDS2. Flanking the recombination hotspots with *attB* and *attP* sequences, the authors demonstrate unidirectional inversion by Bxb1 resulting in *attL* and *attR* sites, suggesting successful repair of the pathogenic allele.

## Programmable Addition Through Site-Specific Targeting Elements

Programmable Addition through Site-specific Targeting Elements (PASTE), developed by Ioannidi et al. (2021), is simply Bxb1 fused to the PE2 prime editor protein (Figure 4B). PASTE achieves integration of DNA segments ranging in size from 779 bp to ∼36 kb at efficiencies up to ∼55% in multiple cell types, including both dividing and non-dividing cells (Ioannidi et al., 2021). The authors observed no off-target activity with PASTE. The range of segment sizes that can be efficiently inserted would enable insertion of more than 99.7% of human cDNAs, illustrating the research potential of the approach. The construct used in PASTE includes a Cas9 protein fused to a reverse transcriptase, a pegRNA, and a large serine integrase Bxb1. Importantly, in a novel step, the construct also incorporates the key elements used for efficient DNA insertion *via* serine integrases, which typically insert sequences containing an *attP* attachment site into a target containing the related *attB* attachment site, or “landing site.” While existing *attP/attB* insertion approaches use a two-step method for DNA insertion, i.e., integration of the *attP* site and associated donor DNA into an *attB* landing site previously incorporated into the genome, the PASTE construct includes both the *attP* site and the *attB* site, the latter of which is incorporated into the pegRNA design and is copied into the genome *via* reverse transcription and flap repair. Because the construct includes both 1) a circular double-strand DNA template containing the donor DNA and the *attP* site, and 2) the *attB* landing site incorporated within the pegRNA design (collectively termed the “attachment site-containing guide RNA; atgRNA”), the DNA cargo can be integrated at the target site in a single reaction. Specifically, the *attB* landing site is inserted *via* a Cas9-directed reverse transcriptase, followed by *attP*-mediated landing site recognition, and integration of the DNA cargo *via* a Cas9-directed integrase.

The authors demonstrated the power and versatility of PASTE by showing that PASTE can be used for: 1) the tagging of genes, i.e., genes were tagged with *GFP* using PASTE, and results showed that *GFP* co-localized with the tagged gene product as expected; 2) multiplexed gene integration, i.e., the authors simultaneously integrated three different genes at three genomic loci; 3) direct insertion of DNA templates carried by AAV or adenoviral vectors; and 4) integration of therapeutic genes with subsequent expression of therapeutic protein products. For example, alpha-1 antitrypsin (encoded by SERPINA1) and carbamoyl phosphate synthetase I (encoded by CPS1) are involved in human Alpha-1 antitrypsin deficiency and CPS1 deficiency, respectively. To test protein production of these two proteins, Ioannidi et al. (2021) used PASTE to deliver SERPINA1 or CPS1 cargo and found effective integration at the ACTB locus in human cells. Furthermore, the authors provided evidence for protein expression, intracellular accumulation of the transgenic products, and secretion of proteins into the medium. Lastly, the authors also took steps to optimize PASTE by using metagenomic mining to discover thousands of putative integrase and attachment site combinations, and to engineer multiple novel integrase orthologs with improved activity and reduced attachment-site requirements. Importantly, a different study used PASTE with human cells to achieve precise integration of templates as large as ∼36 kb with ∼10%–20% integration efficiency (Ioannidi et al., 2021).

## Assembly and Delivery of 100’s of kb of DNA

**FIGURE 5:**
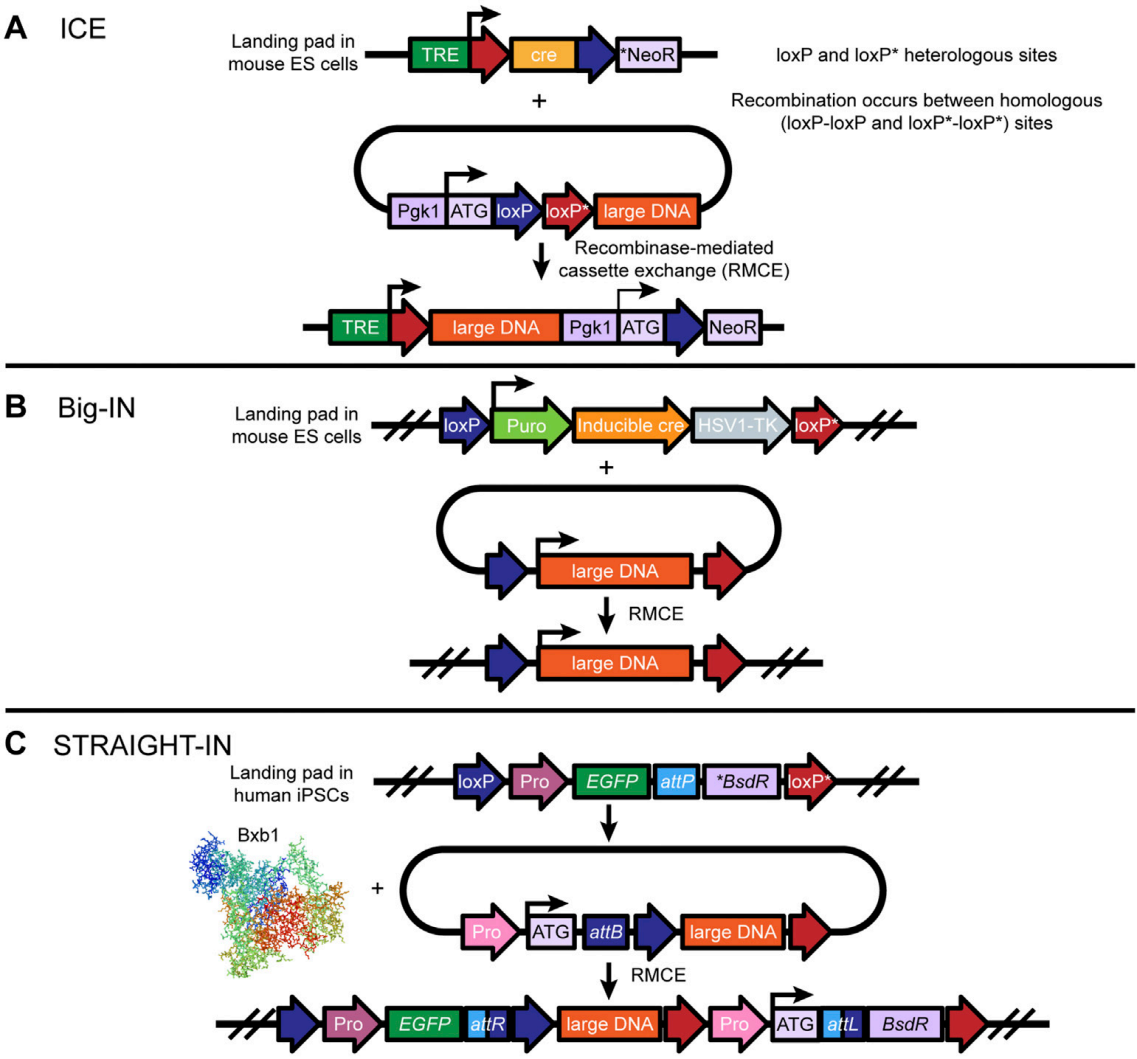
Design and implementation of large DNA transgenesis in mouse ESCs and human iPSCs: **(A)** The ICE (adapted from Iacovino, Bosnakovski, Fey et al.) (Iacovino et al., 2011) tool uses a landing pad site in the safe harbor locus, which contains the cre recombinase gene driven by the tetracycline-responsive promoter (TRE) and an inactive neomycin resistance gene (NeoR) lacking a promoter, a consensus Kozak sequence, and a translation initiation codon (ATG). Notably, only the cre gene is flanked by two heterologous loxP/loxP* sites. Following insertion of the landing pad, large donor DNA flanked by two heterologous loxP*/loxP sites, in the opposite orientation, is integrated into the landing pad *via* doxycycline/tetracycline-induced expression of cre-recombinase. Cre is then replaced by the large DNA, which allows the neomycin resistance gene to be turned on for positive selection of ES cells that have been correctly integrated. **(B)** Big-IN (adapted from Brosh et al.,2021) is also a cre-mediated RMCE-based technique for replacing large sections of mammalian genomes. Using CRISPR/Cas9-mediated HDR, a landing pad site is inserted into a safe harbor locus in mouse ES cells (or human cells) to efficiently engineer mouse and human cells. Insertion of large DNA containing a promoter to drive puromycin, inducible cre, and an HSV1-TK gene, flanked by heterologous *loxP* and *loxP* mutant (loxP*) sites, enables cre-mediated recombination of two pairs of heterologous *loxP* and loxP* sites, resulting in large DNA cassette exchange. While puromycin allows for selection and isolation of a landing pad containing ES cells, correctly integrated ES cell lines can be counter-selected by ganciclovir. Modified ES cells can be taken to germline transmission. **(C)** The Straight-IN (adapted from Grandela et al., 2021) tool uses a combination of Bxb1 (or phiC31)- and cre-mediated recombination to insert a safe harbor locus in human iPSCs for large DNA transgenesis. Similarly to the ICE approach, Straight-IN uses a heterologous loxP and loxP* site-floxed landing pad cassette containing an attP site, an inactive antibiotic resistance gene (*BsdR), and a fluorescent reporter gene driven by a constitutive promoter such as pgk1. The donor vector consists of a cognate *attB* site, a constitutive promoter preceding a translation initiation codon (ATG), and the DNA payload floxed by heterologous loxP and loxP* sites. Successful Bxb1-mediated integration of large DNA results in the formation of attR and attL recombination sites and activation of the antibiotic gene for positive selection of clones. Finally, the double *loxP* and loxP* sites make it possible to remove the selection markers, which are the fluorescent reporter and the antibiotic gene.

Synthetic genomics—the design and synthesis of genomes, or key regions of them—is an emerging field in the study of genome function and biological processes. While synthetic genomics has been applied to investigation of viral and microbial genomes, further advances are required to apply this approach to the study of larger mammalian genomes. Mitchell et al. (2021) developed a strategy that surmounts two major challenges in synthetic genomics: the assembly and delivery of long DNA sequences. Specifically, the investigators developed a workflow termed “eSwAP-IN” (extrachromosomal Switching Auxotrophies Progressively by Integration) that enables *de novo* assembly of DNA sequences of interest in yeast; leveraged a previously described gene-trap-based system termed “ICE” (Inducible Cassette Exchange) to deliver large, assembled DNA constructs to mouse embryonic stem cells (mESCs) (Figure 5A); and tested for payload integration and expression of the integrated gene *via* PCR and immunoblot, respectively. The eSwAP-IN workflow harnesses the inherent capacity of the yeast *Saccharomyces cerevisiae* to perform homologous recombination; *S. cerevisiae* can stitch multiple DNA sequences together with high fidelity, given a minimum of 40 bp of terminal sequence homology encoded by adjacent parts. The eSwAP-IN workflow is a modification of the previously described SwAP-IN method. For “*in yeasto*” DNA assembly, both eSwAP-IN and SwAP-IN incorporate, in a step-wise fashion, DNA segments into a progressively longer construct termed an assemblon. The major modification in eSwAP-IN relative to SwAP-IN is that the assemblon is assembled extrachromosomally in a circular format, and thus replicates and segregates independently of the native yeast chromosomes. Another important advantage of the circular format is that the assemblon can theoretically be directly transferred into *E. coli* for preparation of large quantities of purified DNA for delivery to the organism of choice. Accordingly, the vector used for insertion of the DNA assemblon in the organism of choice encodes features to support replication, segregation, and selection in both yeast and *E. coli*.

To demonstrate the capacity of eSwAP-IN for efficient assembly of large DNA segments, the investigators assembled the 101-kb human *HPRT1* (*hHPRT1*) gene and then delivered the assemblon to mESCs using the ICE system. This system employs an mESC line with a landing pad on the X chromosome that includes a doxycycline-inducible CRE transgene. Induction of Cre expression renders the cells recombination-competent, and delivery of an appropriately designed DNA construct results in cassette exchange recombination and replacement of CRE with the incoming DNA. The investigators demonstrated the use of ICE for successful delivery of a 114-kb construct to mESCs and precise integration of the custom-built 101-kb *hHPRT1* locus into the ICE landing pad.

Although the ICE approach enables delivery of ∼100-kb DNA payloads, it leaves scars flanking the integrated DNA in the mammalian genome. Recently, Brosh et al. (2021) developed an alternative platform, termed Big-IN, for efficient, repeated targeted integration of large DNA segments into mammalian cells, and a scalable pipeline for validation of the engineered cells. They demonstrated use of Big-IN for integration of DNA up to 143 kb in length in human embryonic stem cells (hESCs) and mouse ESCs (Figure 5B).

In brief, a short landing pad is targeted to replace a genomic locus of interest using CRISPR/Cas9-mediated HDR, followed by single-step payload integration *via* Cre recombinase-mediated cassette exchange (RMCE). To accomplish this, cells are transfected with two plasmids: 1) a pCas9 plasmid expressing guide RNAs (gRNAs) targeting the region of interest, and 2) a short landing pad that includes a promotor driving expression of a puromycin-resistance gene, a thymidine kinase gene, and a Cre gene; a mutant lox site (lox 2272) and a loxP site, to permit Cre-mediated RMCE; and, flanking the two lox sites, homology arms (HA) corresponding to the genomic sequences that flank the gRNA target sites at the targeted genomic locus. Fixed insertion of the transiently transfected plasmid is accomplished by inducing its linearization *via* cloning of the same gRNA target sequences and protospacer adjacent motifs into the vector backbone just outside the HAs. Insertion is validated using PCR genotyping with primers targeting the novel junctions between the landing pad and the genomic sequences beyond the HAs; Sanger sequencing for base-pair resolution of correct landing pad integration; and quantitative real-time PCR for loss of target-gene expression and gain of Cre expression. The investigators also developed a modular next-generation sequencing pipeline, including use of hybridization capture sequencing, for validation of loss of the target locus, gain of the landing pad, and absence of the vector backbone and pCas9.

Big-IN is an advance over previous genetic-engineering approaches in that it facilitates one-step scarless delivery of large DNA payloads. Furthermore, the capacity of Big-IN for single-step construct integration enables repeated deliveries to the same allele, thereby minimizing technical factors that can hinder efficient integration, and thus is ideal for comprehensive examination of a given locus. In addition, the approach is designed to be scalable across multiple loci and cell lines, with delivery and selection methods that can be employed in a modular fashion to address problems associated with specific loci and cell types. Further, the validation strategy is designed to enable early validation of construct integration.

Grandela et al. (2021) recently presented STRAIGHT-IN (Serine and Tyrosine Recombinase Assisted Integration of Genes for High-Throughput INvestigation), a novel approach for integrating large DNA payloads into hiPSCs that combines the benefits of both serine (Bxb1) and tyrosine recombinases (cre or Flp) (Figure 5C). The authors first generated hiPSCs with a landing pad cassette containing attachment sites (*attP*) for Bxb1 at the safe harbor locus AAVS1 using CRISPR/Cas9-mediated HDR. Next, using a series of donor DNA constructs ranging in size from 2 to 50 kb with cognate attachment sites (*attB*), the authors examined Bxb1-mediated recombination in hiPSC-landing pad cells and observed efficient (up to 50% with antibiotic selection) integration of donor DNA independent of size restraints. Additionally, to examine the upper limit of DNA payload integration, the authors tested integration of a large 170-kb BAC construct with cognate *attB* sites and observed successful site-specific integration of the large construct into the landing pad site as examined *via* PCR amplification across *attR* and *attL* sites, and *via* digital droplet PCR (ddPCR) to determine the copy number of the integrated DNA. Notably, the landing pad attachment site (*attP*) is flanked by heterologous loxP/loxP* sites, which enables cre-mediated excision of the vector backbone components avoiding any vector-related adverse effects. Together, these findings suggest that the Cas9-Bxb1 toolkit can help with the precise integration of large DNA payloads into hiPSCs as well.

While the aforementioned recombinase-based approaches can theoretically be applied to any gene locus of any length, success depends on the ability to source the required DNA, by PCR or commercial synthesis; the degree to which yeast tolerates the sequence composition of the lengthening assemblon; and the upper limit for chromosome stability in yeast. Mitchell et al. expect that the upper length limit of bacterial or mammalian constructs assembled and maintained in yeast is well over 1 Mb, and that a set of particular technical advances including laboratory automation may facilitate realization of large-scale genome writing in mammalian cells.

## Conclusion and Future Directions

Targeted serine-integrase-based genome insertion is a key component of biomedical research and therapy development. However, until recently, previous approaches lacked the capacity for efficient insertion of large (>5 kb) DNA segments. Recent studies have developed, implemented, and validated multiple versions of a revolutionized Cas9-Bxb1-targeted integrase system to enable diverse novel genetic-engineering approaches based on efficient insertion of large DNA constructs. Future studies can explore the capacity for integration of DNA constructs up to 100 kb in length and further test a “plug-and-play” approach for rapid generation of transgenic mice.

Because serine integrase-based recombination systems exhibit high specificity of for site-directed unidirectional recombination and high orthogonality to the mammalian genome, with no significant off-target activity, these systems have recently received widespread interest. From prophage genomes, using bioinformatics tools, Yang et al. uncovered 34 phage integrases and their predicted *attB* and *attP* recognition sites with no detectible off-target activity. Recently, Durrant et al. developed a systematic computational method for identifying thousands of novel serine integrases and their cognate attachment sites for insertion of large DNA segments. This technique has resulted in identification of three types of serine integrases: 1) integrases that can insert DNA into pre-positioned attachment sites (e.g., Pa01, Si74, and Nm60 integrases showed enhanced recombination of *attP*-donor plasmid DNA into *attB* landing-pad cells with minimal off-target activity compared with either Bxb1 or PhiC31); 2) integrases that can insert DNA into predicted pseudosites (e.g., Sp56, Pf80, and Enc3 can target the human genome at predicted target sites without pre-placed attachment sites); and 3) multi-targeting integrases that can insert DNA into multiple sites simultaneously (e.g., Cp36 integrated DNA into multiple loci with greater than 40% efficiencies in HEK293FT and K562 human cell lines with no pre-positioned landing-pad sites). Furthermore, experimental analyses of these integrases in human cells suggested sevenfold higher integration efficiencies than Bxb1, and genome insertion efficiencies of 40%–70% with DNA insert sizes of 7 kb. Notably, since the computational analyses identified both the integrases and the target sites, this would enable identification of off-targets in the human genome for effective genome therapy.

It will be exciting to apply this technology in cancer genomics, human genetics, systems biology, and cellular engineering to integrate large DNA constructs to model human diseases. Lastly, because the approaches extend beyond the integration of large kb-sized DNA segments to the generation of DNA inversions, it will be interesting to study the efficiency of Bxb1 integrase in generating DNA rearrangements and achieving conditional mutagenesis. In sum, this novel exciting technology, which has been validated both *in vitro* and *in vivo*, has significant clinical implications for treating various human genetic disorders as well as generating and studying next-generation mouse models of human disease. comment

BOX 1 **Site-specific serine recombinases**

Site-specific recombinases are enzymes that cut and rejoin DNA strands at precise genomic locations. Large serine and tyrosine integrases are two classes of bacteriophage recombinases with a nucleophilic active site amino acid residue (serine and tyrosine, respectively) that breaks DNA sequences by targeting DNA phosphodiester linkages (Grindley et al., 2006). Although the functions of tyrosine and serine integrases are similar, their mechanisms of action are very different. Tyrosine recombinases (e.g., Cre and Flp) mediate *bidirectional* DNA integration into the bacterial genome. In contrast, serine recombinases mediate DNA integration into the bacterial genome by catalyzing *unidirectional*, i.e., recombination, between two attachment sites, *attP* (Phage) and *attB* (Bacterium), resulting in the production of recombinant attachment left (*attL*) and right (*attR*) sites (Stark, 2017). Furthermore, compared to tyrosine recombinase attachment sites, the attachment sites of serine recombinases are quite small—50 bp for *attP* and 40 bp for *attB*. Each of these *att* sites (*attP* and *attB*) binds an integrase dimer, and recombination takes place in a complex containing an integrase tetramer that holds two *att* sites (*attL* and *attR*) together. Notably, in conjunction with a serine recombinase, the phage-encoded protein recombination directionality factor (RDF) is required for initiating the reverse reaction, i.e., recombination between recombinant *attL* and *attR* sites in the opposite direction. Because serine recombinases facilitate precise DNA integration, excision, inversion, and translocation, they have been used in a range of applications—molecular genetics, gene therapy, biotechnology, and synthetic biology (Merrick et al., 2018), (Yan et al., 2013)..

BOX 2 **Prime Editing (PE) and Twin Prime Editing (TwinPE)**

Most existing technologies, including those using the CRISPR-Cas9 system, leverage induction of double-strand breaks (DSBs), a process that, as a result of genome damage, results in undesirable outcomes. More recent technologies, including base editing (Komor et al., 2016) and prime editing (Anzalone et al., 2019), circumvent the need for DSBs, but are associated with significant drawbacks. Base editing involves exchanging one nucleotide for another but can be used to make only a small number of nucleotide changes. Prime editing involves cutting only one strand of DNA, followed by the use of reverse transcriptase to “prime,” or initiate, the transfer of new genetic information encoded in an engineered guide RNA termed “prime editing guide RNA; pegRNA,” followed by reconstruction of the other DNA strand so that it corresponds to the new genetic material. While PE has been shown to have the capacity for efficient genome modification, it can generate only small insertions (<∼50 bp).

To enable insertion of DNA sequences larger than ∼50 bp, Anzalone et al. (2021) recently developed a novel twin prime editing (twinPE) strategy. This method uses two pegRNAs—one nicks one of the two DNA target strands, and the other nicks the other strand, with each pegRNA directing the synthesis of a 3′ flap complementary to the 3′ flap produced by the other pegRNA. The 3′ flaps hybridize with each other to form an intermediate strand with annealed 3′ overhangs of the new DNA sequence and annealed 5′ overhangs of the original DNA sequence. After the original DNA sequence is excised, the gap is filled by the reverse transcriptase enzyme, and a nick site ligation is performed between the two nicks, with a 3′ flap sequence replacing the endogenous sequence between the nicks. The edit results in the introduction of a new DNA sequence, either by deleting a portion or modifying a portion of the original DNA sequence. In human cells, twinPE has been shown to successfully generate larger insertions (∼100 bp) than can be generated with the PE strategy. Nevertheless, twinPE falls short of integrating large kilobase-sizes DNA fragments into the genome.
